# XR body illusion for managing pain in fibromyalgia: examining optimal duration

**DOI:** 10.1007/s10055-025-01241-x

**Published:** 2025-12-27

**Authors:** Jennifer Todd, Kirk Woolford, Lee Cheng, Michael C. Lee, Elsje de Villiers, Deanna Finn, Jane E. Aspell

**Affiliations:** 1 School of Psychology, Sport, and Sensory Sciences, Anglia Ruskin University, Cambridge, UK; 2 Centre for Psychological Medicine, Perdana University, Kuala Lumpur, Malaysia; 3 Institute for Creative Technology, Norwich University of the Arts, Norwich, UK; 4 Cambridge School of Creative Industries, Anglia Ruskin University, Cambridge, UK; 5 Department of Medicine, Division of Anaesthesia, University of Cambridge, Cambridge, UK; 6 Department of Physiotherapy, Cambridge University Hospitals NHS Foundation Trust, Cambridge, UK

**Keywords:** Dose finding, Virtual reality, Fibromyalgia, Full body illusion, Cardio-visual, Pain management

## Abstract

**Background:**

Fibromyalgia is a chronic condition characterised by widespread pain, as well as sleep disturbances, fatigue, and memory and concentration difficulties. Research suggests that an alteration in how the brain represents multisensory inputs from the body may cause or maintain chronic pain conditions, including fibromyalgia. Extended reality (XR) and virtual reality setups generating multisensory conflicts have been shown to alleviate pain, however, the optimal duration for such interventions remains unexplored. Here, we aimed to determine an optimal duration for the cardio-visual full body illusion (FBI) in fibromyalgia, considering both tolerability and changes in pain.

**Methods:**

Participants wore headsets to view a video of their own body, filmed from behind, and their virtual body flashed in synchrony with their heartbeat. We used an established dose-finding protocol to determine the ideal duration (balancing benefit and tolerability). Seven cohorts of participants (*N* = 20) were exposed to different durations of the FBI, with adjustments to duration made according to predefined criteria. Measures included a numeric rating scale for pain intensity, pressure pain thresholds, and scales measuring fibromyalgia symptom severity and impact.

**Results:**

We found a quadratic relationship between session duration and changes in self-reported pain-intensity, with 8–16-min durations yielding the most significant improvements. Notably, in the 12-min cohorts pain relief was sustained at 24-h follow-up, and this is the recommended duration for future research.

**Conclusions:**

These findings represent a key step towards developing an effective non-pharmacological intervention for fibromyalgia. Future dose-optimisation research should explore the optimum number of sessions and spacing between sessions.

## Introduction

Numerous studies support the use of extended reality (XR) environments for reducing acute pain during some of the most painful medical procedures (such as wound debridement for burns and episiotomy repair), as well as during labour contractions (e.g., Chan et al. 2018; Malloy and Milling 2010; Xu et al. 2022). Additionally, research suggests that XR can be effective in alleviating experimentally induced pain, with more immersive technology yielding greater pain relief (Malloy and Milling 2010). However, evidence for chronic pain relief with XR is less consistent. While research suggests that XR can reduce chronic pain as long as the patient remains immersed in a virtual environment (Goudman et al. 2022), there is insufficient evidence to support lasting analgesia beyond the immediate post-exposure timeframe with the treatment protocols. The effectiveness of XR for chronic pain may depend on several factors, including the dosage of intervention (longer and more frequent sessions might be needed for lasting effects), the type of equipment used (traditional head-mounted displays may provide more immersion and distraction, but mixed-reality pass-through may lead to greater cognitive engagement), and the specific chronic pain condition being treated.

This paper focuses on the application of an XR intervention for pain management in fibromyalgia (FM), a chronic condition characterised by widespread pain in addition to fatigue, sleep disturbances, and difficulties with memory and concentration (Sarzi-Puttini et al. 2020). The aetiology of FM is not well understood, but research indicates chronic pain conditions, including FM, may arise due to altered multisensory brain representations of the body (Moseley et al. 2012; Tsay et al. 2015). Specifically, scholars have proposed that the “body matrix”—an internal model of the body and surrounding space—is distorted in people with chronic pain and may contribute to the persistence of pain (Moseley et al. 2012). In FM this may manifest as fluctuating body ownership and body boundaries, and heightened attention to internal body signals (e.g., Martínez et al. 2018; Todd et al. 2024; Valenzuela-Moguillansky 2013, Brun 2019).

XR interventions that induce body illusions are theorised to modulate pain in several ways. At a basic level, viewing one’s own body can reduce pain—an effect known as visual analgesia (Longo et al. 2009). Going beyond this, when participants view a virtual body alongside synchronous sensory stimulation, participants experience increased body ownership and a shift in perceived self-location toward the virtual body (Lenggenhager et al. 2007). Some have suggested that such illusions resemble out-of-body experiences which may facilitate dissociation from painful experiences, helping to reduce the affective component of pain (Ramachandran et al. 1998; Pamment and Aspell 2017). Virtual embodiment illusions may also alter multisensory representations of the body (i.e., the body matrix) by introducing multisensory stimuli that challenge and update faulty predictions about bodily inputs (Riva et al. 2019). In doing so, such XR interventions may recalibrate the internal model of the body, restoring a more adaptive body representation and potentially promoting pain relief in a way that is longer lasting than XR interventions that work via distraction (Riva et al. 2019; Matamala-Gomez et al., 2021; Moseley et al. 2012).

Previous research indicates that VR setups designed to induce multisensory conflicts can significantly reduce pain in participants with chronic pain conditions (Álvarez de la Campa Crespo et al. 2023; Pamment and Aspell 2017; Pozeg et al. 2017; Preston and Newport 2011; Preston et al. 2020; Solcà et al. 2018, 2021; Thøgersen et al. 2020; Tong et al. 2020; see also Matamala-Gomez et al. 2019a, 2021 for related reviews). While various body ownership illusion paradigms—including virtual adaptations of the rubber hand illusion—have successfully induced analgesia for localised chronic pain (e.g., Solcà et al. 2018), the widespread (‘full body’) nature of pain in FM means that patients may respond particularly well to XR-presented full body illusions (FBI), which cause changes to the experience of the entire body. In the most commonly used FBI paradigms, participants view a video of their body filmed from behind, while being tapped by an experimenter. When the ‘seen’ and ‘felt’ taps are synchronous, participants report stronger identification with the virtual body and a greater shift in perceived self-location toward it, compared to asynchronous conditions (Lenggenhager et al. 2007). This visuo-tactile FBI reduced pain by up to 37% in a sample of participants with chronic pain conditions (Pamment and Aspell 2017).

FBIs can also be created via cardio-visual synchrony (Aspell et al. 2013). Here, participants view an illuminated silhouette around their virtual body, which flashes synchronously with their heartbeat. As in the visuo-tactile paradigm, the cardio-visual set-up has been shown to increase self-identification with—and self-location toward—a virtual body (Aspell et al. 2013; Heydrich et al. 2018). This FBI has not been examined in relation to pain, but a related illusion—the cardio-visual rubber hand illusion—has been tested in patients with complex regional pain syndrome (Solcà et al. 2018). Here, the illusion reduced pain, improved hand strength, and modulated a physiological pain marker (heart rate variability), supporting the viability of cardio-visual embodiment illusions for chronic pain. We therefore predict that the cardio-visual FBI will reduce pain in patients with FM by altering multisensory body representations.

However, from recent studies, it is not clear what the optimal duration of XR and VR multisensory illusion interventions is for pain relief. Indeed, duration is not always reported, and—where it is reported—there is no consistency within the extant literature, leading to a lack of clarity regarding the significance of this variable. For example, Solcà and colleagues (2018), used 90-s blocks, whereas Pamment and Aspell (2017), used two-minute blocks. In the only identified published XR embodiment study examining people with FM specifically, Świdrak and colleagues (2023) used an FBI, and took precautions to mitigate participant discomfort and fatigue by limiting the total duration of their procedure to 30 min, with the illusion itself lasting approximately 7–8 min. Despite their efforts, it is notable that one participant (from a total of 21) withdrew after completing one session due to physical discomfort, underscoring the importance of considering duration in interventions for pain. It is likely that a minimal duration exists for inducing a sense of ownership for a virtual avatar, which is likely a precursor to analgesia. However, various factors such as the weight of headsets, fatigue, motion sickness and discomfort from certain postures all impact the tolerability of interventions (Tsigarides et al. 2025). Accordingly, it is essential to investigate the potential 'dose–response' relationship between duration and analgesic efficacy.

The main aim of this study was to determine an if there is an optimal duration for the cardio-visual FBI intervention, which balances tolerability and potential pain relief for people with FM. To conduct the study, we modified the dose-finding protocol outlined by Colucci and colleagues (2017). Following this approach, cohorts of three participants were exposed to different durations of the cardio-visual FBI, with adjustments made according to predefined criteria, until an optimal duration was determined. While we are not aware of any literature on which to base a hypothesis regarding optimal duration, we anticipated a starting duration of two minutes would be a tolerable, while providing sufficient immersion (after Pamment and Aspell 2017). We also predicted that the relationship between intervention duration and pain-relief would follow a non-linear pattern, where increasing the duration would initially enhance pain-relieving effects, but that this benefit would plateau or diminish at longer durations as the impacts of waning participant engagement, and discomfort from the headset and seated posture and build up over time (e.g., Świdrak et al. 2023). To test this, we evaluated different functional forms of the dose–response relationship, including both linear and non-linear models. Our primary outcome measure was change in self-reported pain intensity. Additional outcome measures were change in pressure pain threshold—which we expected to increase at optimal durations—and maintenance of pain relief at 24-h follow-up.

## Methods

### The cardio-visual full body illusion

**Fig. 1:**
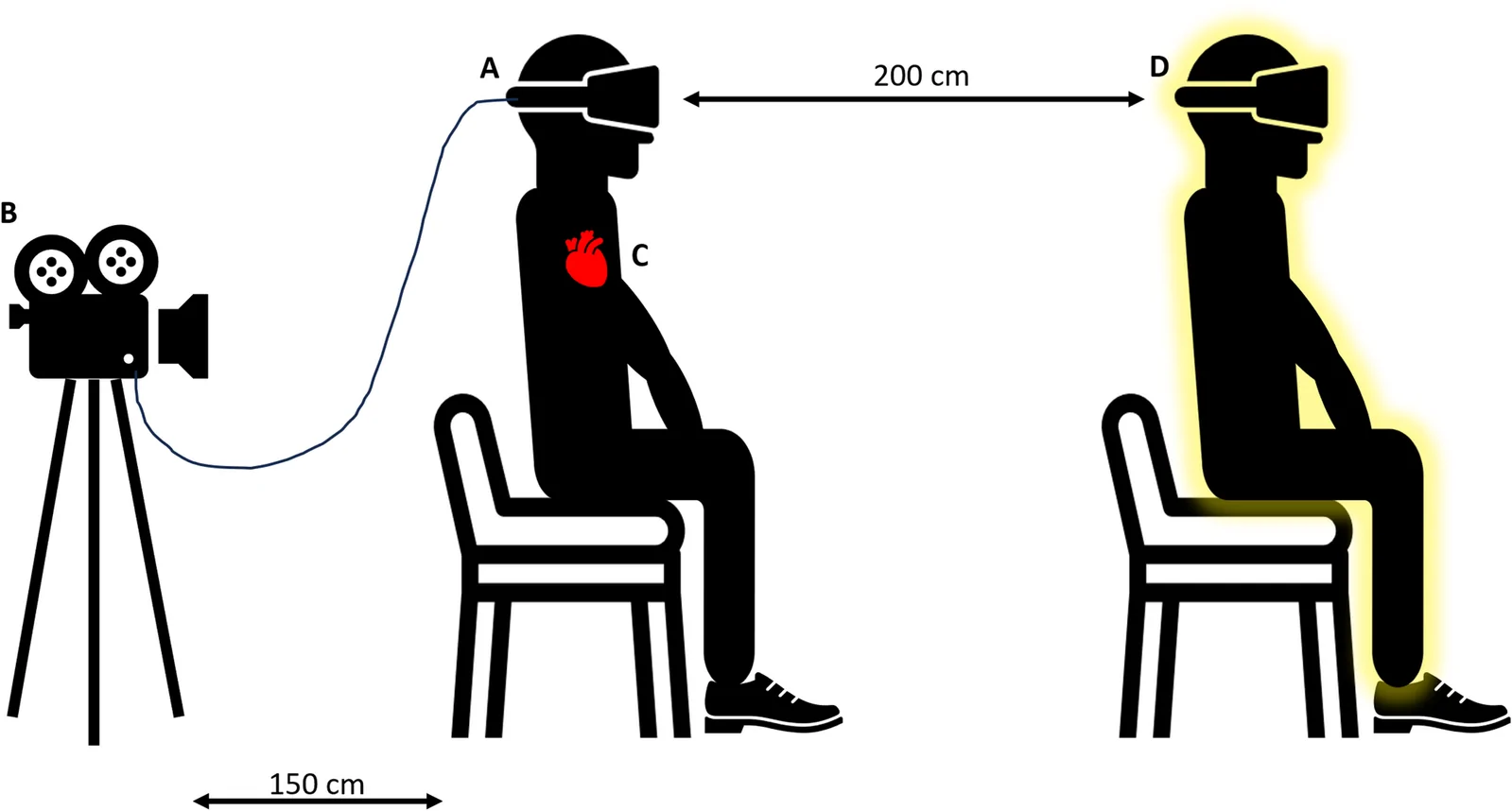
Experimental setup. Participants (A) sat with their backs facing a video camera (B) placed 150 cm behind their stool. The live video showing the participant’s body (their “virtual body”) was projected in real time to the head-mounted display, and sized to appear as though it was sitting 200 cm in front of the participant (D). An electrocardiogram was recorded (C), and R peaks were detected in real time, triggering the participant’s virtual body to visually fade in and out, in synchrony with their heartbeat (D)

A cardio-visual FBI comprising a live video image of the participant’s own body, viewed from behind, was projected into a virtual environment, allowing participants to view their own bodies pulsing in synchrony with their heartbeat (see Fig. 1). Participants were seated instead of standing, as fibromyalgia makes it difficult for many people to stand still for sustained periods. Participants were asked to wear a standalone headset (*Meta Quest 2*). Weighing 470 g. The head mounted display uses an OLED panel with a resolution of 1600 × 1440 pixels per eye display and a refresh rate of 72 Hz. To improve participants’ comfort while wearing the headset, we equipped a strap with battery pack to the headset, which counterbalances the weight of the headset to reduce muscular strain on the neck and shoulders, while flexible braces provide added support. This headset was connected via a USB cable to an Alienware core i9 laptop with an Nvidia RTX3070, running a custom virtual environment, developed in Unity3D.

Unity3D is one of the most widely used video game development platforms and has been officially adopted as the XR application development environment for Apple’s Vision Pro and Microsoft’s HoloLens, and used for numerous medical XR apps including SieVRt, ORamaVR, The Waiting room, etc. However, Unity’s standard templates are optimised for gameplay and user engagement, whereby developers set a target frame rate but the engine attempts to synchronise with the display system, CPU load, and internal processing. The Meta Quest 2 headset’s 72 Hz refresh rate is non-standard and can cause timing slips with the graphic card. App developers normally have no control over when the screen is refreshed, or when code is executed. To have more control over Unity for this experiment, the team wrote a timing thread using Unity’s physics system’s fixed timestep, which refreshed the display at exactly 50 Hz and synchronised image display with heart data as closely as possible.

For image capture and initial video processing we used an *Azure Kinect* camera combining 12-megapixel RGB with 1-megapixel depth camera for body tracking. The Kinect’s depth data was used to separate participants from the background. A spherical (aka 360) image of the room was captured prior to the experiment and used as a backdrop for the virtual environment. This allowed the participant to look around the room, while seeing themselves from the Kinect’s vantagepoint.

Participants were seated on a backless stool positioned 1.5 m in front of the Kinect camera. The camera was positioned to capture participants’ torso and head, along with the stool, from behind. Apart from the headset, three disposable electrocardiography (ECG) electrodes were attached to participants’ chests in the standard three-lead configuration (these were self-attached by participants under their clothes, using a visual diagram). The electrodes relayed QRS output through shielded wires into an eHealth sensor board, producing voltage as they detect the electrical activity of the heart.

Raw ECG was captured via an Arduino Uno Rev3 with a Libelium e-Health sensor board. Custom code was written using the using the *Ardity* library (Wilches 2021) to create an additional timing thread, polling the Arduino/e-Health over USB at 500 Hz and passing incoming raw data signals through a fourth order, zero-phase shift, low-pass Butterworth filter function to normalise and reduce ripple (Bryk 2016). The filter was run for 10 s to balance itself and calculate thresholds for the individual participant’s R-peaks. After the initial priming, the thread checked incoming signals to see if the electrical signal was above the R-peak threshold. If it was, the 1st order derivative (aka slope) between the last signal and current signal were compared to the previous derivative. If the derivative was zero (i.e., tangent), or the derivative changed direction, an R-peak flag was set.

A combination of changing image alpha transparency together with Unity3D particles were used to give participants the illusion of their body image fading in and out, in synchrony with their heartbeats. Once per fixed timestep (i.e., 50 times/sec or every 0.02 secs) the signal thread was checked for an R-peak. If an R-peak had been detected since the last timestep, the image of the participant’s body was set to full opacity, particles were released to create a glowing pulse, and the R-peak flag on the signal thread was reset. If no R-peak was detected in that timestep, the opacity of the body image was reduced by 2%. The particles ran on their own thread and faded over 0.5 secs. This process ensured the flash of the body image was as closely synchronised to the heartbeat as possible within the limits of the system. However, the flash occurs between 20 and 40 ms after the actual R-peak.

From the participant’s perspective, the intervention consisted of sitting on a stool and viewing a real-time video image of their own body, captured from behind and displayed within a 360° image of the laboratory room. This setup allowed participants to look around and feel situated in their actual environment while observing their virtual body pulsing in synchrony with their heartbeat. Participants were free to look around the 3D virtual space during the session, and gently move their arms and torso if so desired, but no specific tasks involving movement were included, as the aim of the intervention was to induce a perceptual full-body illusion via cardio-visual synchrony. Participants were left to sit quietly and were instructed simply to look at the video image, but were advised to tell the experimenter if they felt any adverse symptoms during the intervention (see dose escalation and de-escalation rules, below). They were explicitly told that the visual fading of the avatar was linked to their heartbeat but were not told why this manipulation was used. Between each participant, we used a CleanBox CX1 to disinfect the headset using ultraviolet C light-emitting diodes.

### Evaluation measures

#### The fibromyalgia severity scale (FSS; Wolfe et al. 2016)

The FSS is a self-report questionnaire version of the 2016 American College of Rheumatology FMS criteria (Wolfe et al. 2016) that was designed for clinical and epidemiological research (Wolfe et al. 2010, 2011). The measure comprises a Widespread Pain Index (WPI), where patients indicate whether they experienced pain in the preceding 7 days across 19 different body locations (each indicated body area scores 1 point; maximum WPI score = 19). In the present study, the widespread pain index was assessed using the Michigan Body Map (Brummett et al. 2016). The measure also comprises the Symptom Severity Index (SSI), which assesses the presence and severity of fatigue, waking unrefreshed, and cognitive symptoms in the preceding 7 days (scored from 0 = *no problem’,* to 3 = s*evere: pervasive, continuous, life-disturbing problem*s) in addition to the presence of headaches, pain or cramps in the lower abdomen, and depression in the last 6 months (scored dichotomously: 0 = *no*, 1 = *yes*; maximum SSI score = 12). The WPI and SSI are summed for a total severity score (ranging from 0–31).

Scores from the Fibromyalgia Severity Scale can be used dichotomously to confirm whether a participant meets the ACR 2016 diagnostic criteria for fibromyalgia. For a confirmed diagnosis, participants must: indicate generalised pain (i.e., pain in at least 4 of 5 bodily regions on the WPI; indicate that symptoms have been present at a similar level for at least 3 months; and have a WPI score ≥ 7 and SSI score ≥ 5 OR WPI of 4–6 and SSI score ≥ 9 (Wolfe et al. 2016). Fibromyalgia Severity Scale scores can also be used on a continuous basis to measure the magnitude and severity of fibromyalgia symptoms across participants who satisfy and don’t satisfy the full ACR diagnostic criteria (Wolfe et al. 2016).

#### The small fibre neuropathy questionnaire (SFN; Hoitsma et al. 2011)

The SFN is a 21-item measure that was designed as a screening tool for small fibre neuropathy (Hoitsma et al. 2011). The measure is divided into two sections: the first eight items assess how often participants experience symptoms of small fibre neuropathy (answered on a 5-point scale ranging from 0 = *never* to 4 = *always*), and the remaining items probe how serious the symptoms are (answered on a 5-point scale ranging from 0 = *not at all* to 5 = *seriously*). Overall scores are computed as the sum of all items, and cut-off scores of below 11 indicate no small fibre neuropathy, and above 48 indicate small fibre neuropathy.

#### The pain catastrophizing scale (PCS; Sullivan et al. 1995)

The PCS is a 13-item measure of *catastrophising* (which refers to an exaggerated negative mental state during an actual or anticipated painful experience) which was designed for use in clinical and non-clinical populations (Sullivan et al. 1995). The PCS has a higher-order factor structure, whereby the global construct (general catastrophising) is measured by three related dimensions: rumination (4 items), magnification (3 items), and helplessness (6 items) (Osman et al. 2000). PCS items are rated on a 5-point scale, ranging from 0 (*not at all*) to 4 (*all the time*), and total scores (ranging from 0 to 52) were computed by summing all items, with higher scores indicating greater catastrophising.

#### The 11-point numeric rating scale (NRS) for pain intensity

The NRS for pain is a widely-used single-item measure of pain intensity in adults (Hawker et al. 2011). Although many iterations of the NRS have been reported previously (Hawker et al. 2011), we used an 11-point NRS, where zero represented “no pain”, and ten represented “worst pain imaginable”.

#### The full body illusion questionnaire (FBIQ; Aspell et al. 2013)

To assess the strength of the cardio-visual FBI, we adapted a Full Body Illusion Questionnaire from Aspell et al. (2013), which measures the self-perceived strength of the cardio-visual FBI using a 7-point scale ranging from -3 (*completely disagree*) to 3 (*completely agree*). While the full version of our adapted measure included nine items, the majority were designed as control statements to mitigate demand characteristics (Aspell et al. 2013). The only item that directly assessed illusory body ownership was “I felt as if the virtual body was my body.” Consistent with prior research using full-body illusions (Aspell et al. 2013; Heydrich et al. 2018), we used this item as our primary index of ownership for the virtual body, with higher scores indicating greater ownership/self-identification.

#### Pressure pain thresholds

Pressure pain thresholds (PPTs) were measured via an algometer (Somedic SenseLab, Sweden) which was positioned perpendicular to the trapezius, at the midpoint of the upper border, a ‘trigger point’ commonly used in the establishment of FM diagnosis (Wolfe et al. 1990). Increasing pressure was applied at a rate of 100 kPa/second, and participants were instructed to say “stop” at the first moment that the sensation of pressure changed to one of discomfort (Maquet et al. 2004). The PPT was recorded by the algometer. The mean of three successive measurements was used for the determination of an individual's pressure pain threshold for each of the two sites (left and right; Maquet et al. 2004). The algometer was calibrated following the manufacturer instructions before examining each participant.

### Dose escalation and de-escalation rules

Following the approach of Colucci and colleagues (2017), cohorts of three participants were exposed to varying durations of the cardio-visual FBI, with adjustments made according to predefined criteria (see below) until an optimal duration was determined. The first cohort had a target dose of 2 min, a tolerable and beneficial dose for a similar illusion used by Pamment and Aspell (2017). Target doses for the second and subsequent cohorts were determined in accordance with preset rules and a geometric sequence, whereby the subsequent target dose corresponded to the doubling of the previous dose.

Participants were explicitly advised to tell the experimenter if they felt any adverse symptoms during the intervention, and the experimenter asked participants how they were coping with the intervention at the halfway point of each target dose. A *tolerable duration* was a duration where two or more participants completed the full set duration for that cohort and, at most, only one participant had to terminate their session before the full duration to avoid an adverse consequence (i.e., discomfort, pain, fatigue, or any other adverse symptom, such as dizziness or nausea). *A beneficial duration* was a duration where two or more participants demonstrated an immediate reduction (from baseline to immediately following the intervention) of at least one point on the 11-point Numeric Rating Scale for Pain—the primary outcome measure. The Preset rules were adapted from the protocol outlined by Colucci and colleagues (2017), as follows:

1. If the target dose was tolerable and beneficial for a cohort, the consequent action was to increase the dose for the subsequent cohort.
2. If the target dose was tolerable but not beneficial for a cohort, the consequent action was to increase the dose for the subsequent cohort.
3. If the target dose was not tolerated by two of the three participants, the next dose for the subsequent cohort was decreased.
  - The decrease was 50% of the previous dose increment (e.g., the resultant target dose would be 24 min, if the previous dose had escalated from 16 to 32 min)
  - If applying rule 3a would result in a dose that was longer than the duration at which a previous cohort had terminated their session (indicating that maximally tolerated duration has been identified), the target dose was a determined by calculating 50% increment between the two previously tolerated doses.
4. If a dose was decreased for a cohort and then deemed tolerable and beneficial, the action for the subsequent cohort was to increase the dose. Or, if a target dose which was defined as a 50% increment between two previously tolerated doses was tolerated, a second cohort was recruited to confirm this dose.
5. If a second cohort found any dose tolerable and beneficial, the study was terminated.
6. If after at least one beneficial dose, the subsequent two target doses are tolerable but no further reductions in pain intensity were made, the study was terminated.

### Procedure

Prior to data collection, institutional ethics approval was obtained from the first author’s faculty ethics panel (approval code: PSY-S21-008) on May 27th, 2022. A pilot study was conducted with one participant who has FM to ensure that the methods were suitable. As a result, we determined that participants should be allowed unlimited breaks between tasks during testing sessions, and that participants should be able to complete questionnaires via pen and paper instead of using a computer. Participants were recruited via advertisements in local pain clinics, and via online advertisements with relevant UK pain charities. Inclusion in the study was limited to adults (aged 18 +) who have a previous diagnosis of FM by a qualified healthcare professional, such as a GP or rheumatologist, who have had FM symptoms for at least one year in duration. Exclusion criteria were severe visual impairment, history of severe vertigo, history of motion sickness when wearing headsets, psychosis or body dysmorphia requiring psychiatric treatment, current or planned in-patient hospital treatment during the study, pregnancy, inability to come to the laboratory for a testing session, and inability to provide informed consent. Data were collected between May 2023 and February 2024. Participants first completed an online screening questionnaire to ascertain whether they met the inclusion and exclusion criteria for the study (where FM diagnosis and symptom duration was confirmed by asking participants to report the year that they were diagnosed, and the year that they first started experiencing symptoms), and eligible participants were invited to come to our laboratory for a testing session.

During the laboratory testing session, participants were provided with an information sheet, and had the opportunity to raise any questions or concerns before providing written informed consent. Participants then completed the questionnaire measures detailed above (the FSS, SFN, PCS, NRS and demographic items). Participants also verbally provided information about their fibromyalgia history (years since diagnosis, years since symptom onset, co-morbid conditions, and medication consumption in the preceding 24 h). Next, baseline PPTs were recorded for each shoulder. Participants then experienced the cardio-visual FBI. Participants were assigned to cohorts using a sequential allocation approach. That is, as eligible participants were recruited, they were assigned to the next available dose cohort in order of enrolment. This method ensured balanced progression through the escalating and de-escalating dose conditions without using formal randomisation procedures. The duration each participant achieved was manually recorded using a stopwatch. Immediately following the intervention, participants completed the NRS for pain intensity again, and a second set of PPTs was recorded. Participants then completed the FBIQ, followed by a semi-structured interview about the experience (not reported here). All participants were provided with written debriefing information. Testing sessions lasted between 60 and 90 min, including participant breaks.

24 h after their participation, participants were sent a link to complete the NRS for pain intensity and report any symptoms they had experienced since taking part in the study. Participants were offered a retail voucher worth £16 as remuneration for their time.

### Statistical analyses

Analyses were conducted using JASP (version 0.10.2, JASP team, 2019) and *R* (version 4.4.3, R core team, 2025). There were no missing data. Descriptive statistics were calculated to summarise sample characteristics. The primary outcome variable was change in self-reported pain intensity from t_1_ to t_2_, calculated as the difference between the two scores. Additional outcome variables were maintenance of change in self-reported pain intensity at 24 h (the difference between ratings at t_2_ and t_3_), and change in PPT from t_1_ to t_2_, analysed separately for each shoulder.

To evaluate different potential functional forms of the dose–response relationship, we examined the fit of linear regression models and quadratic regression models, using intervention duration as the predictor, and change in pain intensity from t_1_ to t_2,_ or change in PPT from t_1_ to t_2_, as criterion variables, respectively. Model comparisons between the linear and quadratic fits were conducted using likelihood ratio tests. Where neither model explained a significant proportion of variance, paired-samples *t*-tests were used to assess overall pre–post changes across all durations. Effect sizes were estimated using Cohen’s *d* (small = 0.2, medium = 0.5, large ≥ 0.8; Cohen 1988), with 95% confidence intervals.

Body ownership ratings, indexed via a single item from the FBIQ, were examined as a mechanism, rather than an outcome. We examined dose–response relationship on an exploratory basis to test whether intervention duration influenced body ownership ratings. We also explored whether illusion strength was associated with change in pain intensity ratings using Pearson’s correlation coefficient, where *r*—0.10 = was considered weak, 0.30 medium, and 0.50 strong (Cohen 1988).

Finally, we conducted an exploratory ANCOVA to assess whether the effect of intervention duration on post-intervention pain intensity remained significant after adjusting for baseline pain. This was motivated by an observed trend toward higher baseline pain in the 12-min cohorts. Intervention duration was entered as the independent variable, post-treatment pain as the dependent variable, and pre-treatment pain as a covariate. Pairwise comparisons of estimated marginal means were computed with Bonferroni correction. Given the small sample size and non-randomised cohort assignment, this analysis was treated as exploratory and interpreted with caution.

## Results

### Sample characteristics

In total, six cohorts of three participants, and one cohort of two participants were recruited (*N* = 20, all women), which is sufficient for a dose finding study (Colucci et al. 2017; Tighiouart and Rogatko 2012). Participants were aged between 26 and 71 (*M* = 52.50, ± 10.23 years; for more detailed breakdown by cohort, see Table 1). The majority of participants (*n* = 16) reported that they were White British (British Black or African Caribbean *n* = 2; mixed or multiple ethnic groups *n* = 1; prefer not to say *n* = 1). Regarding educational attainment, six participants had achieved A-Levels, one had a diploma, four had an undergraduate degree, seven had a post-graduate degree, and two had some other qualification. Finally, eight participants were retired, four were unemployed, three were employed part-time, two were employed full-time, one was self-employed full-time, and one was self-employed part-time, and one was on long-term sick-leave.

**Table 1:** Sample characteristics for the total sample, and for each of the seven cohorts respectively

	All cohorts	Cohort 1	Cohort 2	Cohort 3	Cohort 4	Cohort 5*	Cohort 6	Cohort 7
Age in years, *M* (range)	52.50 (26–71)	56.33 (53–58)	54.33 (49–59)	47.67 (26–69)	51.67 (49–56)	50.00 (48, 52)	50.00 (29–71)	56.67 (47–62)
Widespread Pain Index, *M* (range)	11.40 (5–19)	9.33 (5–14)	10.67 (6–14)	13.67 (7–19)	10.00 (9–12)	12.5 (6, 19)	16.33 (12–19)	7.67 (5–11)
Symptom Severity Index, *M* (range)	8.15 (3–12)	9.67 (8–11)	8.67 (6–11)	6.33 (3–8)	7.00 (5–8)	8.5 (5, 12)	9.33 (8–11)	7.67 (5–10)
ACR criteria met, *n*	16/20	2/3	2/3	2/3	3/3	1/2	3/3	3/3
Small Fibre Neuropathy Questionnaire, *M* (range)	62.00 (36–83)	64.00 (58–71)	56.67 (51–62)	56.33 (36–83)	62.00 (55–66)	61.50 (50, 73)	73.67 (61–82)	59.67 (52–65)
Pain Catastrophising Scale, *M* (range)	26.40 (2–47)	30.67 (13–43)	30.00 (16–47)	22.00 (12–32)	26.33 (15–41)	28.00 (14, 42)	27.00 (13–37)	21.33 (2–34)

^*^Cohort 5 comprised two participants instead of three, because the target dose was not tolerated by either participant

Participants self-reported that they had been living with FM symptoms for between 1 and 40 years (*M* = 15.18 years, ± 10.23 years), and had been diagnosed between 1.5 and 40 years ago (*M* = 6.33 years, ± 5.38). Four participants were just below thresholds for the ACR FM self-report diagnostic criteria for research on the day of testing (Wolfe et al. 2016; see Methods, and Table 1), based on slightly deflated scores on either the widespread pain index or the symptom severity index. However, we elected to include these participants on the basis that all participants indicated that they had been formally diagnosed with FM by a general practitioner or rheumatologist, and given the known symptom variability associated with FM (e.g., Bartley et al. 2018; Okifuji et al. 2011). 17 of the 20 participants were using pharmacological treatments for their pain, and had consumed medication within a 24-h period prior to the testing session. Participants reported a range of comorbidities, with several individuals reporting more than one condition. The most frequently reported were osteoarthritis (*n* = 6), anxiety (*n* = 5), rheumatoid arthritis (*n* = 4), myalgic encephalomyelitis/chronic fatigue syndrome (*n* = 4), and endometriosis (*n* = 4).

### Dose benefit and tolerance

Following the pre-set rules, the duration was escalated up to a target of 32 min. However, participants were not able to achieve this duration (aborting at 18-min and 22-min). The maximal tolerated dose was 16 min, and a target dose of 12 min was examined (rule 3b) and confirmed as beneficial in two cohorts (rule 5), at which point the study was terminated.

**Fig. 2:**
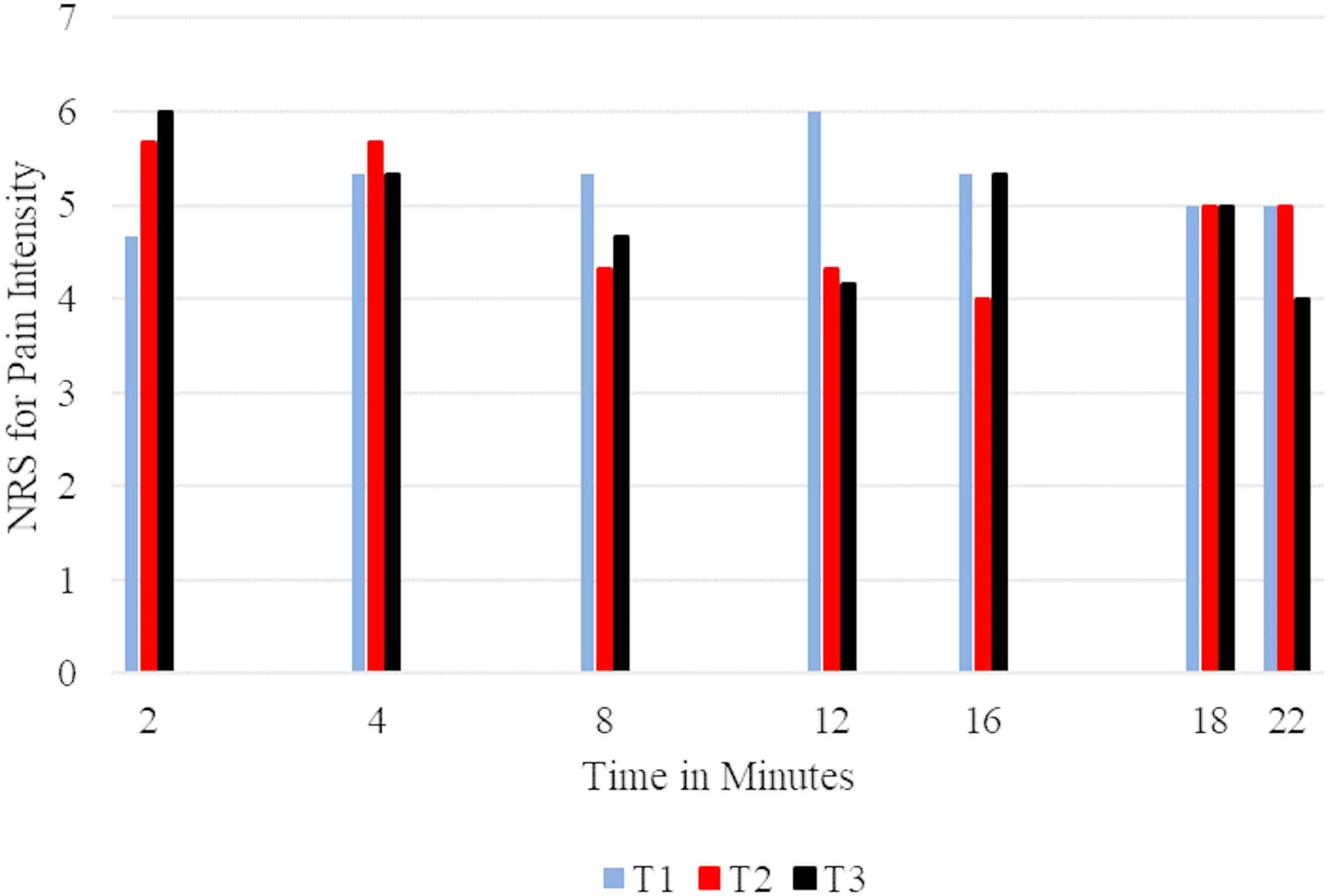
Average scores on the Numeric Rating Scale (NRS) for pain intensity for each cohort at each of the three time points, where t_1_ = pre-intervention, t_2_ = immediately post-intervention, and t_3_ = 24 h post-intervention

The 2-min, 4-min, and 32-min doses were not beneficial, with participants experiencing either no change in pain intensity, or an increase in pain intensity from t_1_ to t_2_, and at 24-h follow-up (t_3_, see Table 2). The 8-min, 12-min, and 16-min doses all resulted in decreases in pain intensity from t_1_ to t_2_, and these changes were sustained or partially sustained at t_3_ in the 8- and 12-min cohorts (but not the 16-min cohort). Overall, participants in the 12-min cohorts demonstrated the greatest decreases from t_1_ to t_2_ (*M* = -1.67), and pain reduction was maintained from t_2_ to t_3_ (*M* = -0.17; see Fig. 2), suggesting that 12 min should be identified as the recommended duration for future research.

**Table 2:** Individuals’ adherence to target dose, per cohort, with decisions made on feasibility and efficacy as per the primary outcome measure, and data for additional outcome measures

Cohort	Participant	Target Dose	Dose Achieved	Dose Tolerable	NRS T_1_	NRS T_2_	NRS T_3_	Dose beneficial	PPT left t_1_	PPT left t_2_	PPT right t_1_	PPT right t_2_	FBIQ	t_3_ symptoms
1	001	2 min	Yes	Yes	8	8	9	No	103.67	103.00	84.33	110.33	-3	Headache, neck pain, fatigue
	002		Yes		3	3	4		163.00	162.33	142.33	157.67	3	none
	003		Yes		3	6	5		92.33	93.67	116.67	95.67	3	Headache, shoulder pain, fatigue
2	004	4 min	Yes	Yes	7	8	7	No	144.33	180.33	153.67	170.67	1	Headache, neck pain, fatigue
	005		Yes		5	5	4		201.00	276.00	217.00	292.33	-3	None
	006		Yes		4	4	5		218.33	308.33	266.00	320.33	-2	Headache
3	007	8 min	Yes	Yes	7	7	8	Yes	220.33	192.33	108.33	160.00	1	Fatigue
	008		Yes		4	3	3		341.33	408.67	249.33	315.67	-3	None
	009		Yes		5	3	3		72.00	101.00	62.67	113.33	-3	Headache
4	010	16 min	Yes	Yes	5	3	4	Yes	164.33	184.67	163.00	198.67	-2	None
	011		Yes		6	5	5		199.67	228.33	175.33	226.67	-2	Fatigue
	012		Yes		5	4	7		119.33	140.00	98.00	138.33	-3	Headache, neck pain
5	013	32 min	22 min 12 s	No	5	5	4	No	243.00	261.67	348.67	351.67	-3	None
	014		18 min 03 s		5	5	6		427.33	369.33	343.33	364.67	0	Shoulder pain
6	015	12 min	Yes	Yes	6	5	4	Yes	58.00	66.67	51.67	75.00	-2	None
	016		Yes		8	6	5		27.67	29.00	23.33	28.33	-1	Headache, neck pain, fatigue
	017		Yes		7	6	3		75.00	107.67	97.00	106.67	1	Stiffness, fatigue
7	018	12 min	Yes	Yes	7	4	1	Yes	115.00	123.00	156.67	162.67	-3	None
	019		Yes		3	1	7		72.00	62.67	83.00	60.33	3	Neck pain, fatigue
	020		Yes		5	4	5		117.67	123.33	123.00	126.33	-3	Fatigue

NRS = Numeric Rating Scale for Pain Intensity, PPT = Pressure Pain Threshold, FBIQ = Full Body Illusion Questionnaire Item 2 scores for self-identification with the virtual avatar, t_1_ = pre-intervention, t_2_ = immediately post-intervention, t_3_ = 24 h post-intervention

### Dose–response relationship

#### Self-reported pain intensity

**Fig. 3:**
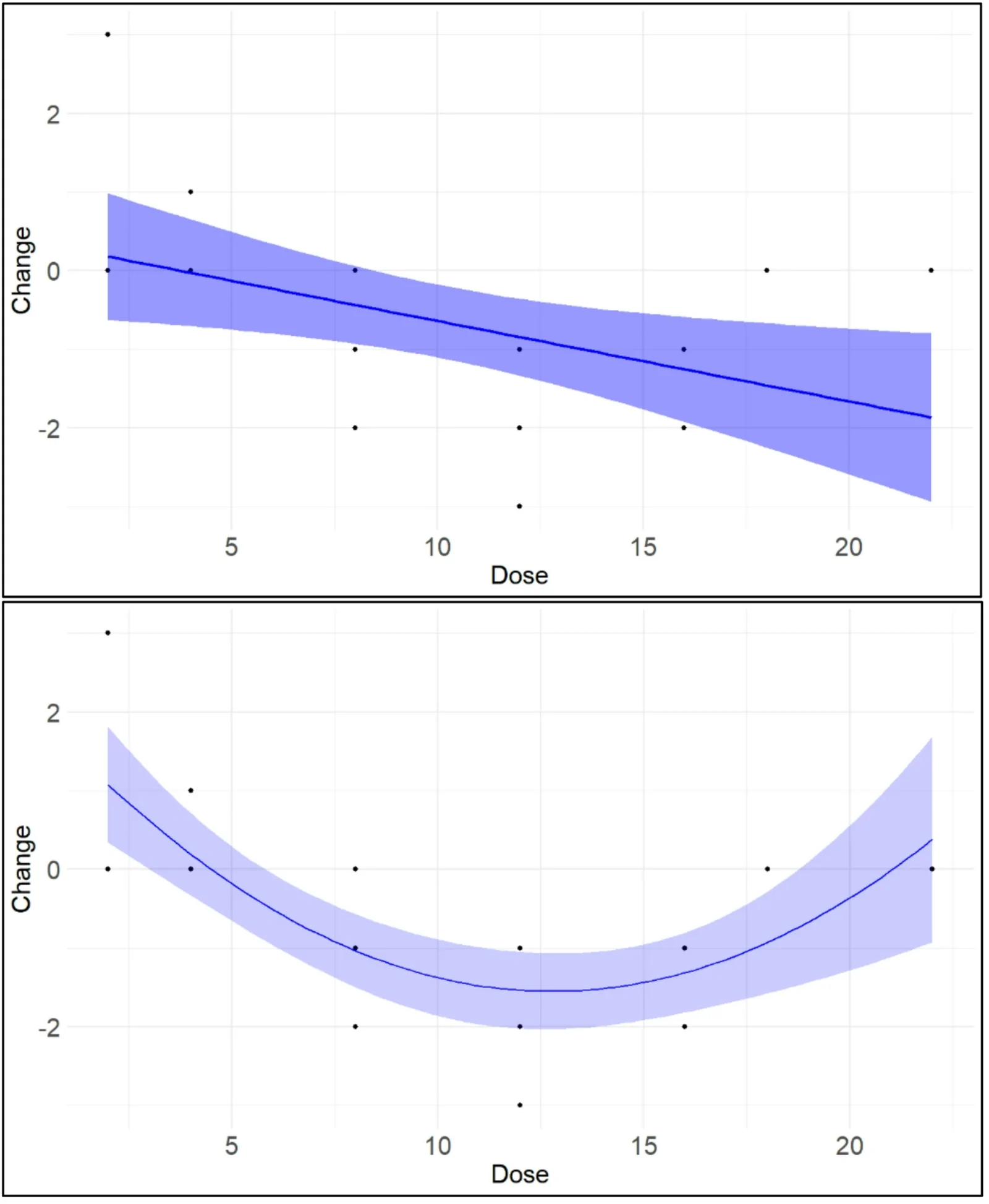
Linear (top) and quadratic (bottom) dose–response relationships for changes in scores on the Numeric Rating Scale for pain intensity from baseline (t_1_) to immediately following the intervention (t_2_). A positive integer indicates that pain increased across the two timepoints, and a negative integer indicates that pain decreased. The trendline depicts the linear/quadratic equations, and the shaded areas depict 90% confidence intervals. Note that for some cohorts, two or more participants had the same change scores, so fewer datapoints can be seen

We first examined a linear model of the relationship between change in pain intensity and duration (see Table 3). A statistically significant model was identified, whereby increased intervention duration was associated with decreased change in pain intensity (i.e., reduced benefit), but the model only accounted for 17% of the variance in the data, (see Fig. 3). Next, we examined the hypothesised quadratic model (see Table 3). Here, a statistically significant model was identified, which accounted for a 52% of the variance, whereby change in self-reported pain intensity from t_1_ to t_2_ = 0.02(duration)^2^—0.58(duration) + 2.14. (see Fig. 3). Comparison of the models indicated that the quadratic model had significantly improved model fit compared to the linear model, χ^2^(1) = 12.37, *p* < 0.001.

**Table 3:** Model summaries for the linear and quadratic models of the dose–response relationship for pain intensity, pressure pain thresholds, and self-identification

Outcome measure	Model		*R*^2^	Adj. *R*^2^	*F*(df)	*p*	B	95% CI	SE	*t*	*p*
Change in pain intensity	Linear		.21	.17	4.78 (1,18)	.042	-0.10	-0.21, -0.01	.05	2.19	.042
	Quadratic	dose	.57	.52	11.46 (2,17)	< .001	-0.58	-0.85, -0.31	.13	4.46	< .001
		dose^2^					0.02	0.01, 0.04	.01	3.82	.001
Change in pressure pain threshold (left shoulder)	Linear		.09	.04	1.69 (1,18)	.210	-1.70	-4.39, 1.05	1.31	1.30	.210
	Quadratic	dose	.09	.02	0.82 (2, 17)	.459	-0.79	-11.18, 9.60	4.92	0.16	.874
		dose^2^					-0.04	-0.52, 0.43	0.22	0.19	.851
Change in pressure pain threshold (right shoulder)	Linear		.02	.04	0.34 (1,18)	.567	-0.63	-2.90, 1.64	1.08	0.58	.567
	Quadratic	dose	.04	.07	0.34 (2, 17)	.717	1.68	-6.86, 10.21	4.05	0.41	.684
		dose^2^					-0.11	-0.50, 0.28	0.19	0.59	.561
Self-identification	Linear		.03	.03	0.51 (1, 18)	.483	-0.06	-0.25, 0.12	0.09	0.72	.483
	Quadratic	dose	.09	.02	0.79 (2, 17)	.468	-0.39	-1.08, 0.30	0.33	1.19	.250
		dose^2^					0.01	-0.02, 0.05	0.02	1.04	.315

##### Post hoc analysis: adjusting for baseline pain

While the 12-min dose was associated with the greatest reductions in pain and strongest maintenance at follow-up, the 8-min and 16-min doses also showed benefit. As shown in Fig. 2, the 12-min cohort appeared to have higher pre-intervention pain intensity. Although there were no statistically significant differences in baseline pain between cohorts (*p* = 0.921), this trend raised the possibility that the analgesic effect in this group might reflect baseline variability rather than dose-specific effects. To explore this, we conducted an exploratory ANCOVA controlling for pre-intervention pain. Although the small sample size limits statistical power and interpretability, baseline pain was a strong independent predictor of post-intervention pain (*F*(1,13) = 40.08, *p* < 0.001, η^2^ = 0.56). This analysis also revealed a significant main effect of duration on post-intervention pain intensity after adjusting for baseline pain, (*F*(5,13) = 3.71, *p* = 0.026, η^2^ = 0.26). Post-hoc comparisons of estimated marginal means (adjusted for pre-intervention pain) using Bonferroni correction showed that the 12-min dose group reported significantly lower post-treatment pain than the 2-min group (mean difference = –2.60, SE = 0.72, *p*_*bonf*_ = 0.049). No other pairwise comparisons reached statistical significance.

#### Pressure pain thresholds

Data for each shoulder were treated separately. Neither the linear model (which accounted for 4% of the variance in the data), nor the quadratic model (which accounted for 2% of the variance in the data) supported a statistically dose–response relationship for changes in pressure pain thresholds on the left shoulder (see Table 3), and there was not a statistically significant difference in model fit between the models, χ^2^(1) = 0.04, *p* = 0.836. Similarly, for the right shoulder, neither the linear model (which accounted for 4% of the variance in the data), nor the quadratic model (which accounted for 7% of the variance in the data), supported a statistically dose–response relationship for changes in pressure pain thresholds (see Table 3), and there was not a statistically significant difference in model fit between the models, χ^2^(1) = 0.41, *p* = 0.523. Because dose–response relationships were not identified, we examined differences in pressure pain thresholds from t_1_ to t_2_ across all durations (*N* = 20) using paired samples *t*-tests. For the left shoulder, participants tended to demonstrate higher pressure pain thresholds at t_2_ (*M* = 176.10 ± 103.96), compared to t_1_ (*M* = 158.77 ± 98.84), a statistically significant difference of 17.33 (95% CI = 33.22, 1.45), *t*(19) = 2.29, *p* = 0.034, corresponding to a medium effect size (*d* = 0.51, 95% CI = 0.97, 0.04). Similarly, for the right shoulder, participants tended to demonstrated higher pressure pain thresholds at t_2_ (*M* = 178.77 ± 100.74), compared to t_1_ (*M* = 153.17 ± 90.79), a statistically significant difference of 25.60 (95% CI = 38.31, 12.89), *t*(19) = 4.22, *p* < 0.001, corresponding to a large effect size (*d* = 0.94, 95% CI = 1.46, 0.40).

#### Body ownership

Neither linear regression analysis (which accounted for 3% of the variance in the data), nor analysis of a quadratic relationship (which accounted for 2% of the variance in the data), supported a statistically dose–response relationship for body ownership scores (see Table 3), and there was not a statistically significant difference in model fit between the models, χ^2^(1) = 1.22, *p* = 0.269. There were moderate, positive correlations between self-identification scores and pain change from t_1_ to t_2_ (r = .42, *p* = .068), and from t_1_ to t_3_ (r = .42, *p* = .062), whereby greater self-identification was associated with larger changes in self-reported pain intensity. However neither correlation was statistically significant.

## Discussion

In this study we sought to determine the optimum duration for an XR cardio-visual FBI intervention that was balanced in terms of tolerability and benefit for participants with FM. In terms of our primary outcome measure—change in self-reported pain intensity—we identified a significant dose–response relationship that broadly aligned with our hypotheses. That is, we identified a quadratic relationship where the shortest and longest durations provided no benefit, while participants who experienced the mid-range durations (8—16 min) experienced the greatest reductions in pain intensity. We identified a duration of 12-min as the most promising duration for further research, with the largest average reduction in pain and effects maintained at 24-h follow-up. While exploratory in nature, an ANCOVA controlling for baseline pain confirmed a significant main effect of duration, and revealed that the 12-min group reported significantly lower post-treatment pain than the 2-min group. This pairwise comparison provides preliminary evidence that 12 min may be more effective than very short exposures. In contrast, pressure pain thresholds were not predicted by duration, but were significantly higher after the intervention compared to baseline when data were collapsed across durations, with medium and large effect sizes identified for the left and right shoulders, respectively.

In broad alignment with previous literature indicating that XR body ownership illusions can significantly reduce pain in participants with chronic pain conditions (Matamala-Gomez et al. 2019a, b; Pamment and Aspell 2017; Pozeg et al. 2017; Preston and Newport 2011; Preston et al. 2020; Solcà et al. 2018, 2021; Thøgersen et al. 2020; Tong et al. 2020), we found that the cardio-visual FBI can reduce pain intensity for participants with FM, and significantly increase pressure pain thresholds. The present study did not include an asynchronous control condition, and therefore cannot conclusively attribute the analgesic effects to the multisensory illusion itself. However, our finding of moderate, positive correlations between ‘illusion strength’ (responses to the ownership question in the illusion questionnaire) and change in pain intensity provides some indication that the pain-relieving effects are at least partly related to the illusion. It is possible that reductions in pain were also partly driven by more general mechanisms such as distraction, which may influence pain perception by capturing finite attentional resources and diminishing the conscious experience of painful sensations. That is, XR interventions compete for attention that would otherwise be directed towards pain stimuli, potentially resulting in increased pain thresholds and reductions in self-reported pain intensity (Goudman et al. 2022; Mallari et al. 2019).

Visual analgesia—the phenomenon whereby viewing one's own body can reduce pain—may have also contributed to the observed effects (Longo et al. 2009). Additionally, previous work has proposed that FBIs may elicit a form of out-of-body experience, which may facilitate detachment from the physical body and reduce the affective dimension of pain (Pamment and Aspell 2017; Ramachandran et al. 1998). This also corresponds with studies that have examined neurologically induced out-of-body experiences, where an intense sense of disembodiment has been associated with decreased pain intensity (Bunning and Blanke, 2005). One hypothesis is that the effectiveness of virtual body ownership illusions may be, in part, due to the fact that they create a multisensory conflict which can modulate body ownership, and—potentially—disrupt or recalibrate the distorted body matrix implicated in chronic pain (Matamala-Gomez et al. 2021). This theoretical framework aligns with the moderate, positive correlations we identified between ownership over the virtual body and changes in pain intensity, although these correlations were not statistically significant. These findings provide tentative support for the possibility that the analgesic effects may be related not only to seeing one's body, but also to specific aspects of the illusion, such as changes in bodily self-consciousness underpinned by changes in multisensory body representations. We suggest that these changes may be responsible for the prolonged pain relief demonstrated, which XR interventions based on distraction may not achieve. Nonetheless, future research including asynchronous sham conditions and immersive VR control groups will be essential to isolate these specific mechanisms.

Consistent with our hypothesis, a starting duration of two minutes was tolerable. However, contrary to previous literature, neither this duration nor the four-minute duration were beneficial in terms of reducing pain intensity. In previous research a two-minute FBI reduced pain in a sample with mixed chronic pain diagnoses (Pamment and Aspell 2017), and 90-s sessions of a cardio-visual rubber hand illusion reduced pain and increased heart rate variability in participants with complex regional pain syndrome (Solcà et al. 2018). However, there are notable differences between the paradigms used in the present study and previous studies. The FBI used by Pamment and Aspell (2017) relied on visuo-tactile congruency, as opposed to the synchronous cardio-visual input used in the present study. It is possible that visuo-tactile input induces experiences of the FBI more rapidly or intensely, perhaps due to the perceptual salience of tactile input compared to the cardiac input, which could explain the discrepancy between the two studies. However, this idea remains speculative because no research to date has directly compared the cardio-visual and visuo-tactile FBIs. Similarly, the cardio-visual rubber hand illusion used by Solcà and colleagues (2018) is more localised than the cardio-visual FBI used in the present study, as it is only applied to one arm. It is possible that shorter induction periods may be sufficient to induce pain relief for body-part ownership illusions (compared to FBIs), however future research comparing the cardio-visual FBI and hand illusions is needed to test this hypothesis.

Further methodological distinctions between the present and previous studies exist, notably in the composition of the samples. In the present study, participants had widespread pain from FM, whereas the sample reported by Solcà and colleagues (2018) comprised adults with upper limb complex regional pain syndrome, and the sample used by Pamment and Aspell (2017) comprised a majority diagnosed with localised chronic pain. It is conceivable that participants with more widespread pain, such as those with FM, may need to experience body ownership illusions for longer durations to derive potential benefits, particularly given the disruptions in embodiment in FM (e.g., Martínez et al. 2018; Todd et al. 2024), however further research is needed to examine this possibility. The differences between the present study and previous findings emphasise the need for further efforts to standardise methodological approaches in body illusion research to better understand who will benefit from them.

The maximal tolerated dose in the present study was 16-min; participants in the 32-min target dose cohort aborted at 18-min and 22-min, respectively. Based on the present findings, we can conclude that beyond 16-min, the benefits are outweighed by the potential negative impacts of wearing the headset (which can feel heavy) and adopting the requisite seated posture (e.g., possible neck pain, shoulder pain, and fatigue). Examination of the dose–response relationship indicated that 12-min is the optimal dose for use in future research. Participants in the 12-min cohorts experienced an average decrease in self-reported pain intensity of 1.67 (measured via the 11-point numeric rating scale) immediately after the intervention compared to baseline. These participants also experienced an additional average decrease in self-reported pain intensity of 0.17 at 24-h follow-up (compared to immediately following the intervention), suggesting that reductions in pain intensity were maintained for at least 24 h following the intervention.

However, it is still possible that—for some participants—the recommended dose will not be suitable, since, as noted by Świdrak and colleagues (2023), an intervention lasting 7–8 min caused one participant with FM (from a total of 21) to withdraw due to discomfort. While the sample size in the present study is sufficient for a dose finding study (Colucci et al. 2017; Tighiouart and Rogatko 2012), future dose-articulation research is needed to evaluate and extend the present findings (Craig et al. 2008; Dalton et al. 2021). In particular, future research should seek to confirm the present findings in a new cohort, and examine further dose dimensions such as scheduling (i.e., the impact of the number of sessions and temporal spacing between sessions) to identify the optimal dose regimen for FM (Dalton et al. 2021). Future studies should also include longer-term follow-up (e.g., up to seven days) to assess the longevity of analgesic effects beyond the immediate and short-term timeframe, and how this may interact with scheduling.

### Limitations

While this study presents several novel findings, these should be interpreted in the context of some limitations. First, the dose–response relationship identified in the present study may be specific to the *Meta Quest 2* headset (e.g., see Tsigarides et al. 2025) and the cohort utilised (i.e., predominantly white women). While women are more likely to be affected by fibromyalgia (Sarzi-Puttini et al. 2020), it is possible that a different optimal dose exists for men, and future research considering this intervention in more diverse samples is warranted. Future research should also include a thorough examination and characterisation of FM characteristics (e.g., quantitative sensory testing profiles), which was beyond the scope of the present study. A more detailed characterisation will provide further insights into who may benefit from the intervention, and who may not. In addition, we did not collect data on socio-economic status in the present work, and this may also be an important variable to consider in future research, as there may be demographic disparities in the access to XR-based treatments.

While our inclusion criteria specified adults aged 18 and over, our sample included a wide age range (26–71 years), and age was not stratified across cohorts. Although core fibromyalgia symptoms appear broadly consistent across adulthood, previous studies have reported small but significant age-related differences: some research indicates that younger individuals tend to report slightly higher symptom impact scores (Cronan et al. 2002; Jiao et al. 2014), while other studies suggest that older adults show poorer physical function than younger adults, but broadly similar symptom severity (Di Carlo et al. 2022, Mottelson et al., 2023). Relatedly, participants reported living with fibromyalgia symptoms for between 1 and 40 years. This variability reflects the real-world heterogeneity of fibromyalgia, where symptom onset, diagnosis, and illness trajectory can vary widely across individuals. It also likely reflects the broad age range discussed above (age was moderately correlated with symptom duration). Future studies with larger samples should seek to stratify or model age and/or symptom duration to explore their potential role as moderators of treatment response.

Additionally, while all participants had a prior diagnosis of FM, many also reported comorbid health conditions, including other rheumatic diseases (e.g., rheumatoid arthritis, osteoarthritis) and endometriosis. Importantly, these were not mutually exclusive groups: several individuals had multiple comorbidities, reflecting the clinical complexity commonly seen in FM populations (Häuser et al. 2015; Weir et al. 2006; Häuser et al. 2019). Although this heterogeneity reflects real-world patient presentations and enhances ecological validity, it also introduces variability that may have influenced pain responses. Again, future studies should seek to stratify or control for common comorbidities to explore whether they moderate responses to XR-based embodiment interventions.

There are challenges in identifying minimally important clinical differences for chronic pain cohorts, with factors such as baseline pain and methodological differences impacting estimates (Olsen et al. 2018). Accordingly, at present, it is unclear whether the changes in self-reported pain intensity based on the numeric rating scale for pain, or the changes in pressure pain thresholds, are clinically significant for an FM cohort (Maquet et al. 2004; Olsen et al. 2018). Finally, while we demonstrated that the cardio-visual FBI is beneficial for reducing pain intensity, is also important to note that participants did report experiencing some additional symptoms at 24-h follow-up, including headaches, neck pain and fatigue. It is not possible to know in the present study whether these symptoms are due to the intervention, or sit within participants’ normal pattern of FM symptoms, and this should be a further explored in future dose-articulation research, particularly when examining the impact of scheduling (Dalton et al. 2021), and the longevity of pain relief following the intervention.

Further research is also warranted to investigate the neural mechanisms underlying the pain-relieving effects induced by the cardio-visual FBI in FM. Brain imaging studies are necessary to investigate neural changes occurring during and/or after the intervention. Such studies would provide insights into the neurological processes associated with the intervention's efficacy and could inform additional future treatment strategies for FM. Further research could also seek to incorporate more comprehensive assessments of embodiment, such as standardised multi-dimensional questionnaires (e.g., Roth and Latoschik 2020), to better characterise participants’ subjective experience of the illusion and its relationship to therapeutic outcomes.

### Conclusions

These limitations notwithstanding, the present study provides novel evidence of a significant dose–response relationship for the cardio-visual FBI in terms of pain intensity reduction for participants with FM, with mid-range durations (8–16 min) showing the greatest efficacy. We recommend a 12-min duration for use in future research, considering both efficacy and participant tolerability. The present study also provides novel evidence that the cardio-visual FBI increases pressure pain thresholds in FM, and this effect was not linked to session duration. We also observed moderate, positive correlations between self-identification with the virtual avatar and changes in pain intensity, although these correlations were not statistically significant. These findings provide tentative support for the possibility that analgesic effects may go beyond the effects of seeing one's own body, and may be related to specific aspects of the illusion that facilitate body ownership of the virtual body, that is, multisensory integration of synchronous visual and cardiac signals. While our findings largely align with previous literature on body ownership illusions and pain reduction, the duration-specific finding highlights the need for more research using standardised approaches in this research domain. Overall, future dose-articulation research considering the cardio-visual FBI is warranted, and this should further explore dose dimensions such as scheduling and examine long-term pain relief sustainability.

## Data Availability

The author confirms that all data analysed during this study are included in this published article.
